# TREM2 and microglia exosomes: a potential highway for pathological tau

**DOI:** 10.1186/s13024-022-00581-5

**Published:** 2022-11-17

**Authors:** Nimansha Jain, Jason D. Ulrich

**Affiliations:** 1 Department of Neurology, Washington University School of Medicine, St. Louis, MO USA; 2 Hope Center for Neurological Disorders, Washington University School of Medicine, St. Louis, MO USA; 3 Knight Alzheimer’s Disease Research Center, Washington University School of Medicine, St. Louis, MO USA

## Abstract

Tau pathology appears to spread along neuronal networks via the template misfolding of tau by pathological tau conformations. The mechanisms underlying neuron-to-neuron transmission of tau are unclear and recent work demonstrates a role for microglia in the spread of tau pathology. In this Commentary, we discuss a recent study that found that loss of TREM2 expression resulted in exacerbated spread of tau pathology that depended on microglial exosomes. These important findings highlight the role of the microglial endolysosomal system and TREM2 in the spread of tau pathology.

## Main text

The development of tau pathology is tightly correlated with neurodegeneration and cognitive decline in Alzheimer’s disease (AD). Aggregates of insoluble, hyperphosphorylated tau occur not only in AD, but also in neurodegenerative diseases including frontotemporal dementia, Pick’s disease, chronic traumatic encephalopathy, corticobasal degeneration, and progressive supranuclear palsy. Pathological tau appears to propagate along functionally connected neuronal networks via pathological tau conformations inducing templated misfolding and aggregation of monomeric tau. Both neuronal and non-neuronal mechanisms mediate the spread of tau pathology. Although the molecular mechanisms are still poorly understood, several lines of evidence indicate that synaptic activity promotes the release of tau from neurons into the extracellular space as either free protein or within exosomes, which are secreted vesicles derived from multivesicular bodies (MVBs). How neurons take up extracellular tau is also not well understood, although binding to heparin sulfate proteoglycans and LRP1 have recently been shown to play an important roles in neuronal tau endocytosis [1].

**Fig. 1:**
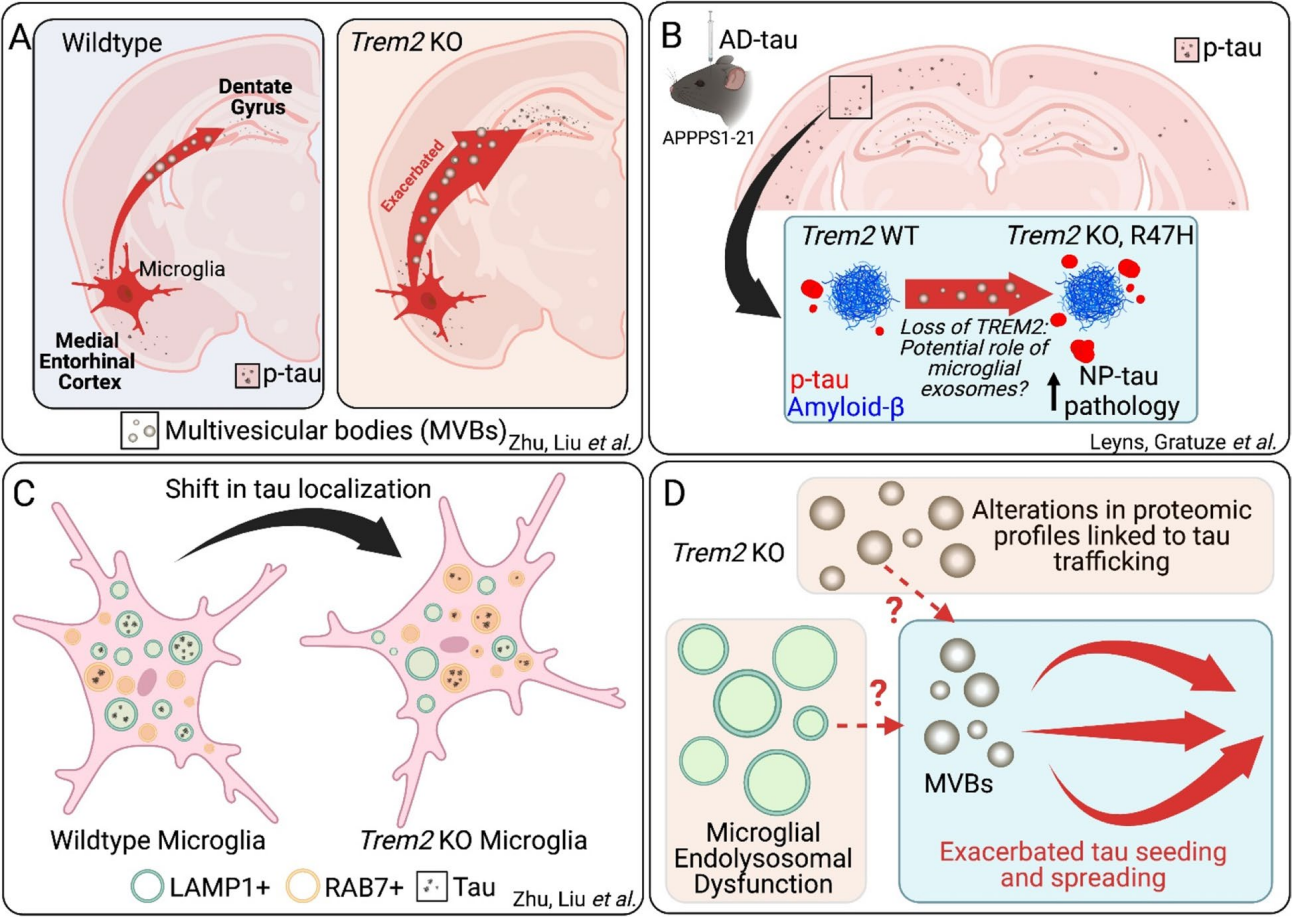
Effects of TREM2 and microglia on exosomal spread of tau. **A** *Trem2* KO mice exhibit exacerbated spread of tau through exosomes into the dentate gyrus region of the hippocampus from the medial entorhinal cortex using AAV expression of P301L tau. **B** *Trem2* KO and R47H mice exhibit increased neuritic plaque tau pathology in APPPS1–21 mice seeded with insoluble tau-enriched AD brain lysate (AD-tau). **C*** tau* localization shifts from from LAMP1+ vesicles to RAB7+ vesicles in *Trem2* KO microglia. **D** Underlying questions on the role of *Trem2* in endolysosomal function and proteomic profiles that may be mechanistically affecting multivesicular bodies and tau trafficking pathways

Microglia may be an important intermediary in facilitating the spread of pathological tau between neurons. Although microglia do not express tau mRNA, microglia isolated from the rTg4510 tauopathy mouse model or from human AD or primary tauopathy cases contained tau protein [2]. When primary microglia from tauopathy patients or tauopathy mouse models were cultured they released tau in the media, which could in turn induce tau aggregation in cellular assays [2]. Expression of P301L human tau in the medial enthorinal cortex (MEC) of wild-type mice using AAV led to the spread of tau pathology to the dentate gyrus 1 month post-infection [3]. Depleting microglia from the brain using CSF1R antagonists inhibited the spread of AAV-expressed P301L tau from the MEC to the hippocampus, as did inhibition of exosome synthesis or release [3]. How microglial-associated genetic risk factors for AD, such as TREM2 or APOE4, affect microglia’s ability to spread tau remains unclear. TREM2 KO results in increased p-tau seeding around amyloid plaques in amyloid mouse models following injection of insoluble tau-enriched AD brain lysate (Fig. 1) [4]. However, the relative contribution of TREM2-dependent, microglial-mediated tau spread versus increased neuritic dystrophy around plaques is not known.

A recent study by Zhu, Liu and coworkers investigated the role of TREM2 in tau spreading from the MEC to the hippocampus using AAV expression of P301L tau (Fig. 1) [5]. In contrast to how removal of microglia inhibited the spread of tau pathology, TREM2 KO mice exhibited exacerbated spread of tau into the dentate gyrus region of the hippocampus, which also corresponded with synaptic loss and cognitive impairment. Using a three-chamber cell culture system, the authors demonstrated that microglia were necessary intermediaries in the spread of tau between non-adjoining neurons. Mice injected with exosomes produced by TREM2 KO microglia incubated with oligomeric, recombinant human tau exhibited greater levels of p-tau staining after 21 days than mice injected with exosomes from TREM2 WT microglia. TREM2 KO-derived exosomes also exhibited greater seeding activity in a cellular tau seeding assay. The observed increase in tau spreading adds to a complicated body of literature surrounding the role of TREM2 in tau pathology. In some tau mouse models, such as the PS19 model that overexpresses P301S human tau or a P301L model, TREM2 KO did not have a striking effect on tau pathology, and in fact reduced neurodegeneration in the PS19 model [6]. In contrast, in the hTau mouse model that expresses all six isoforms of human tau in place of mouse tau, TREM2 KO resulted in increased tau phosphorylation. It is possible that differences in the aggressiveness of tau pathology across these different tauopathy models may explain the variance in effects of TREM2 on tau phosphorylation.

How TREM2 influences microglial extrusion of tau in exosomes remains to be resolved. Microglial or macrophage uptake of tau does not appear to depend on TREM2 expression in vitro. However, TREM2 appears to regulate the intracellular itinerary within the endolysosomal system. Zhu, Liu et al. found that 4 hours after oligomeric tau administration, tau in WT microglia robustly co-localized with LAMP1+ lysosomes. However, in TREM2 KO microglia, the subcellular distribution of tau shifted away from LAMP1+ lysosomes and towards Rab7+ late endosomal compartments (Fig. 1). A previous study found TREM2 KO bone marrow-derived macrophages (BMDMs) did not degrade insoluble tau enriched from AD brain as effectively as TREM2 WT BMDMs using a pH-sensitive fluorescent tag, consistent with an impairment in trafficking of tau to lysosomes [7]. More generally, iPSC-derived microglia that were homozygous for the Nasu-Hakola-associated p.Q33X mutation and lacked functional TREM2 protein exhibited impaired lysosomal acidification and protein degradation with accumulation of non-degraded protein in MVBs, similar to the accumulation of tau in CD63+ MVBs observed in TREM2 KO microglia [8]. Lysosomal impairment disrupted degradation of material within MVBs leading to expulsion of MVB contents via exosomes [9]. Pharmacological disruption of lysosomes in SH-SY5Y cells overexpressing α-synuclein led to increased seeding-competent α-synuclein release in exosomes [10]. Given these results, it will be interesting to test whether a TREM2-dependent lysosomal impairment contributes to the increase in tau release in microglial exosomes.

Overall, Zhu, Liu et al. describe how loss of TREM2 function could potentiate the spread of tau pathology, which could in part explain how TREM2 variants affect AD risk. The results provide new mechanistic insight into how TREM2 and microglia may influence p-tau seeding and spreading around amyloid plaques. In addition to understanding how TREM2 KO increases the release of tau in microglial exosomes, it will be important to further investigate whether and how microglia modify tau to influence its seeding potential. Targeting microglial dispersion of tau is an exciting potential therapeutic avenue for mitigating the spread of pathological tau throughout the brain to slow the relentless progression of tauopathies.

## Data Availability

Not applicable.

## Abbreviations

AD: Alzheimer disease
MEC: Medial entorhinal cortex
AAV: Adeno-associated virus
MVB: Multivesicular body
BMDM: Bone marrow-derived macrophage
TREM2: Trigger receptor expressed on myeloid cells 2
APOE: Apolipoprotein E
LAMP1: Lysosomal associated membrane protein 1
